# Urinary TWEAK as a biomarker of lupus nephritis: a multicenter cohort study

**DOI:** 10.1186/ar2816

**Published:** 2009-09-28

**Authors:** Noa Schwartz, Tamar Rubinstein, Linda C Burkly, Christopher E Collins, Irene Blanco, Lihe Su, Bernard Hojaili, Meggan Mackay, Cynthia Aranow, William Stohl, Brad H Rovin, Jennifer S Michaelson, Chaim Putterman

**Affiliations:** 1 Division of Rheumatology, Albert Einstein College of Medicine, 1300 Morris Park Avenue, Bronx, NY 10461, USA; 2 Hadassah University Hospital, POB 12000, Ein Kerem, Jerusalem 91120, Israel; 3 Biogen Idec, 14 Cambridge Center, Cambridge, MA 02142, USA; 4 Department of Medicine, Division of Rheumatology, Los Angeles County + University of Southern California Medical Center and University of Southern California, Keck School of Medicine, 2011 Zonal Avenue, Los Angeles, CA 90033, USA; 5 Washington Hospital Center, 110 Irving Street, NW, Washington, DC 20010, USA; 6 Feinstein Institute for Medical Research, 350 Community Drive, Manhasset, NY 11030, USA; 7 Ohio State University Medical Center, 410 West 10th Avenue, Columbus, OH 43210, USA; 8 Department of Microbiology and Immunology, Albert Einstein College of Medicine, 1300 Morris Park Avenue, Bronx, NY 10461, USA

## Abstract

**Introduction:**

TNF-like weak inducer of apoptosis (TWEAK) has been implicated as a mediator of chronic inflammatory processes via prolonged activation of the NF-κB pathway in several tissues, including the kidney. Evidence for the importance of TWEAK in the pathogenesis of lupus nephritis (LN) has been recently introduced. Thus, TWEAK levels may serve as an indication of LN presence and activity.

**Methods:**

Multicenter cohorts of systemic lupus erythematosus (SLE) patients and controls were recruited for cross-sectional and longitudinal analysis of urinary TWEAK (uTWEAK) and/or serum TWEAK (sTWEAK) levels as potential biomarkers of LN. The performance of TWEAK as a biomarker for nephritis was compared with routinely used laboratory tests in lupus patients, including anti-double stranded DNA antibodies and levels of C3 and C4.

**Results:**

uTWEAK levels were significantly higher in LN patients than in non-LN SLE patients and other disease control groups (*P *= 0.039). Furthermore, uTWEAK was better at distinguishing between LN and non-LN SLE patients than anti-DNA antibodies and complement levels, while high uTWEAK levels predicted LN in SLE patients with an odds ratio of 7.36 (95% confidence interval = 2.25 to 24.07; *P *= 0.001). uTWEAK levels peaked during LN flares, and were significantly higher during the flare than at 4 and 6 months prior to or following the flare event. A linear mixed-effects model showed a significant association between uTWEAK levels in SLE patients and their disease activity over time (*P *= 0.008). sTWEAK levels, however, were not found to correlate with the presence of LN or the degree of nephritis activity.

**Conclusions:**

High uTWEAK levels are indicative of LN, as opposed to non-LN SLE and other healthy and disease control populations, and reflect renal disease activity in longitudinal follow-up. Thus, our study further supports a role for TWEAK in the pathogenesis of LN, and provides strong evidence for uTWEAK as a candidate clinical biomarker for LN.

## Introduction

Renal involvement in systemic lupus erythematosus (SLE), known as lupus nephritis (LN), is a common and serious complication, with reports of 5-year renal survival with treatment ranging from 46 to 95% [1]. LN is characterized by a relapsing- remitting course, requiring constant follow-up and surveillance and often entailing changing treatments. A number of biochemical markers are currently used to clinically assess patients' disease activity, such as anti-double-stranded DNA antibodies (anti-dsDNA Abs) and complement component levels. Nevertheless, the correlation between these markers and LN is imperfect, and their utility in reflecting disease activity and in predicting outcome remains controversial [2].

TNF-like weak inducer of apoptosis (TWEAK) is a cytokine that has drawn much attention since its initial identification in 1997 [3]. TWEAK, and its cognate receptor Fn14, are TNF/TNF receptor superfamily members, respectively, which have been found to be involved in many physiological processes, such as cellular proliferation [4,5], migration [6], survival [7], differentiation [8], and induction of apoptosis [3,9-11]. TWEAK/Fn14 interactions have also been found to induce inflammation as they upregulate a number of chemokines, cytokines and adhesion molecules in various tissues [12,13]. While TWEAK and Fn14 genes are widely expressed, their expression level is low in normal tissues but is dramatically elevated in the context of injury and disease [14]. Currently it is thought that TWEAK facilitates physiologic tissue repair and regeneration following acute injury, but in the setting of chronic inflammatory diseases the dysregulated expression of TWEAK is pathogenic [14,15].

Previously we reported that urinary TWEAK (uTWEAK) levels can reflect LN activity [16]. In the present paper we present results of further studies performed to elucidate the relationship of uTWEAK to human SLE and LN, and the possible clinical uses of measuring TWEAK levels. To that end, we have cross-sectionally analyzed uTWEAK levels in a multicenter cohort of SLE patients with and without documented renal disease, as well as across several control populations. In addition, a longitudinal analysis of a prospectively followed group of LN patients was performed in order to track uTWEAK levels in individual patients, thereby assessing the ability of uTWEAK to serve as a clinical marker of disease exacerbation and remission. We also explore the possible use of other TWEAK measurements, including serum TWEAK (sTWEAK) levels and the sTWEAK/uTWEAK ratio.

## Materials and methods

### Patients

The present study was based on three cohorts of patients, all previously described in detail: the Albert Einstein College of Medicine (AECOM) lupus cohort, based on patients followed regularly at lupus clinics in the Jacobi Medical Center and the Montefiore Medical Center, Bronx, NY, USA [16]; the Ohio SLE Study (OSS), Columbus, OH, USA [17]; and the University of Southern California patient cohort, including patients admitted to the Los Angeles County + University of Southern California Medical Center and seen in consultation by the Rheumatology Service or seen as outpatients at the Rheumatology Clinics of Los Angeles County + University of Southern California Medical Center in Los Angeles, CA, USA [18]. The studies at all participating institutions were approved by their respective institutional review boards. Informed consent was obtained from all patients participating in the present study. All enrolled patients fulfilled at least four of the 1982 revised American College of Rheumatology criteria for the diagnosis of SLE [19]. In the three cohorts, all LN patients had undergone a kidney biopsy confirming their renal disease histologically, while all non-LN SLE patients never had documented renal involvement.

Besides routine clinical laboratory tests performed at the time of each visit, each patient provided a freshly voided morning urine specimen and/or blood sample. Urine samples were centrifuged to remove sediment, and serum was aliquoted from centrifuged blood samples before being frozen at - 80°C.

Two separate studies were performed in this investigation: the first was a cross-sectional study of both uTWEAK and sTWEAK in LN patients and controls, while the second was a longitudinal study of uTWEAK levels in SLE patients over time.

The cross-sectional uTWEAK study included only SLE patients from the AECOM cohort: 30 biopsy-proven LN patients with active renal disease at the time of the visit and 49 non-LN SLE patients. In addition to the SLE patients, four groups of controls were analyzed: healthy controls, 28 individuals with no known history of SLE or any other kidney or autoimmune disease recruited from a Jacobi Medical Center obstetrics and gynecology clinic as well as volunteers from the staff of AECOM; renal controls, 31 patients with kidney disease due to diabetes (n = 15) or hypertension (n = 16) recruited from Jacobi Medical Center and Montefiore Medical Center nephrology clinics; 79 rheumatoid arthritis (RA) patients, recruited from Jacobi Medical Center and Montefiore Medical Center rheumatology clinics; and 25 osteoarthritis (OA) patients, also recruited from Jacobi Medical Center and Montefiore Medical Center rheumatology clinics.

The cross-sectional sTWEAK analysis was performed based on patients and controls from the above-described AECOM cohort who had serum samples drawn at the time of their clinic visit. Overall, 23 LN patients, 43 SLE non-LN patients, 133 disease controls and 19 healthy individuals were analyzed. In addition, serum samples of 35 LN patients and 31 non-LN SLE patients from the University of Southern California cohort were analyzed for sTWEAK, as well as serum from 20 healthy control subjects recruited from personnel of the Los Angeles County + University of Southern California Medical Center and the University of Southern California Keck School of Medicine.

The second study was longitudinal, based on data from 13 LN patients in the OSS cohort. In this cohort, LN subjects were considered as such only after LN was confirmed by a kidney biopsy, in addition to these patients having had two or more SLE flares that required an increase in immunosuppressive therapy within the past 3 years or having had 4 months of disease activity despite therapy. The 13 analyzed patients are unique in that they had a documented visit in which they were undergoing a LN flare (defined below), in addition to having regular visits in the months prior to and following the flare event. A systematic examination of changes in uTWEAK levels before, during and following the flare was therefore possible.

In a separate analysis, these OSS patients were combined with 31 SLE patients from the AECOM cohort (18 patients with LN and 13 patients without evidence of nephritis) on whom longitudinal data were available, to examine the relationship between uTWEAK levels and disease activity over time.

### Classification of systemic lupus erythematosus activity status

LN activity was evaluated based on a subset of the Systemic Lupus Erythematosus Disease Activity Index (SLEDAI) 2000 [20], designated the renal Systemic Lupus Erythematosus Disease Activity Index (rSLEDAI). The rSLEDAI consists of the four kidney-related items of the SLEDAI 2000, including hematuria (>5 red blood cells/high-power field), pyuria (>5 white blood cells/high-power field), proteinuria (>0.5 g/24 hours or urine protein/creatinine ratio >0.5) and urinary casts (heme, granular, or red blood cell). The presence of each of the parameters is scored as 4 points; the renal activity score can therefore range from 0 to 16. For our inclusion criteria, any rSLEDAI score >0 was considered as active LN, unless otherwise specified.

Systemic lupus activity was scored with the original SLEDAI 2000. OSS patients were additionally classified regarding the presence and severity of a renal flare, based on predefined increases in urine sediment, serum creatinine levels and proteinuria from their baseline levels, as described previously in detail by Rovin and colleagues [17,21].

### TWEAK measurement

TWEAK levels in serum and urine were determined by ELISA, as described previously [16]. Briefly, microtiter plates were coated with the BEB3 murine monoclonal anti-TWEAK antibody [7] in bicarbonate buffer overnight. The plates were then blocked with 3% BSA/PBS for 6 hours, washed, and urine samples diluted 1:3 or serum samples diluted 1:15 in 3% BSA/PBS were added in duplicate. Serial dilutions of recombinant soluble human TWEAK [7] were also added to the plates, enabling construction of a standard curve. Following an overnight incubation at 4°C, the plates were washed, and a solution of pre-mixed biotinylated murine anti-TWEAK antibody P5G9 [13] and avidin- horseradish peroxidase was added for 1 hour at room temperature. Finally, the plates were washed and a developer solution was added. TWEAK levels were derived from an average of the duplicate assays that were carried out for all samples. All assays were performed blindly, without knowledge of the patient's identity, disease presence or disease activity.

### Assay standardization

To take into account variations in urine concentration, uTWEAK levels were corrected to urine creatinine, when levels of the latter were available. Corrected uTWEAK levels are therefore expressed as picograms per milligram of creatinine (pg/mgCr); otherwise, uTWEAK levels, as well as sTWEAK levels, are expressed as picograms per milliliter (pg/ml).

The C3, C4 and anti-dsDNA Ab measurements obtained at different centers and measured in different laboratories were standardized by dividing the value received for each patient by the mid-normal range of the measuring laboratory.

### Statistical analysis

The data were not normally distributed, so median values and interquartile ranges were calculated as measures of central tendency; the data are therefore expressed as the median (interquartile range), unless otherwise indicated. The Mann- Whitney *U *test was used to compare between two groups and the Kruskal- Wallis test was utilized for comparing three or more groups. The Kruskall-Wallis test was followed by Dunn's post-hoc testing. Proportions were compared by the two-sample test of proportions. Association among categorical variables was measured by Pearson's chi-squared test. Correlations were performed using the Spearman rank correlation coefficient, followed by Sidak adjustment for significance levels to account for multiple comparisons.

Area under the curve (AUC) calculations of nonparametric receiver operating characteristic (ROC) curves were used to compare the ability of the various biomarkers to distinguish between specific groups of patients. In addition, sensitivity and specificity characteristics were derived from the ROC curves and were used to identify cutoff point values for defining high and low TWEAK levels.

Logistic regression was performed on the cross-sectional data, and the odds ratio was derived. Longitudinal data from the OSS were log-transformed and analyzed using repeated-measures analysis of variance, followed by Dunn's post-hoc testing for nonparametric data; these data were graphed using a least-squares means plot. A linear mixed-effects model was constructed based on longitudinal data from the OSS and AECOM cohorts to examine the relationship between TWEAK levels and patients' disease activity over time. *P *< 0.05 was considered significant. The statistics programs used for the analysis were Intercooled Stata version 9.2 (StataCorp LP, College Station, TX, USA) and GraphPad Prism version 4.03 (GraphPad Software, San Diego, CA, USA).

## Results

### Urinary TWEAK is a marker of lupus nephritis

The demographic characteristics of the different groups included in this cross-sectional analysis of uTWEAK are presented in Table 1. In all groups but the renal controls, more than 85% of the individuals were women. As might be expected, the RA patients, OA patients and renal controls were older than both SLE patient groups and healthy individuals. Creatinine levels in the lupus and control groups were as follows: LN patients, 0.85 (0.6 to 1.2); SLE non-LN patients, 0.7 (0.6 to 0.8); RA patients, 0.8 (0.7 to 0.9); OA patients, 0.8 (0.7 to 0.9); and renal patients, 2.3 (1.7 to 3.3). Patients in the RA group did not have associated renal involvement, as seen by their normal creatinine levels, and the normal urinalyses obtained around the time of the sample for uTWEAK in the large majority of RA patients for which these were performed. As compared with the patients with LN, the renal control group had significantly higher creatinine levels (*P *< 0.001), while those in the SLE non-LN group were lower (*P *= 0.004). The LN and non-LN groups, however, were similar in terms of age, sex and ethnicity.

**Table 1:** Cross-sectional study demographics

	LN patients	Non-LN patients	Normal controls	Rheumatoid arthritis patients	Osteoarthritis patients	Renal controls	*P *value all (LN vs. non-LN)
Number of patients (% female)	30 (93)	49 (88)	28 (89)	79 (90)	25 (84)	31 (55)	- (0.474)
Age (years)	34.5 (28 to 44)	41 (34 to 46)	37 (29 to 45)	58 (51 to 67)	63 (57 to 77)	63 (56 to 67)	<0.001* (0.074)
Ethnicity^a^	14/14/0/2	27/17/0/2^b^	7/14/3/4	-	-	-	-
African American (%)	46.6	58.7	25	-	-	-	- (0.304)
SLEDAI	10 (7 to 16)	3.5 (2 to 6)	-	-	-	-	- (<0.001*)
SLEDAI without renal component	2 (0 to 6)						- (0.426)

Continuous variables presented as the median (interquartile range). LN, lupus nephritis; SLEDAI, Systemic Lupus Erythematosus Disease Activity Index. ^a^African American/Hispanic/Caucasian/other.^b^Ethnicity of three additional non-LN systemic lupus erythematosus patients is unknown. *P < 0.05. The Mann- Whitney *U *test was used to compare two groups with continuous variables, while the Kruskal- Wallis test was used for three or more groups. The two-sample proportion test was used for categorical variables.

**Figure 1:**
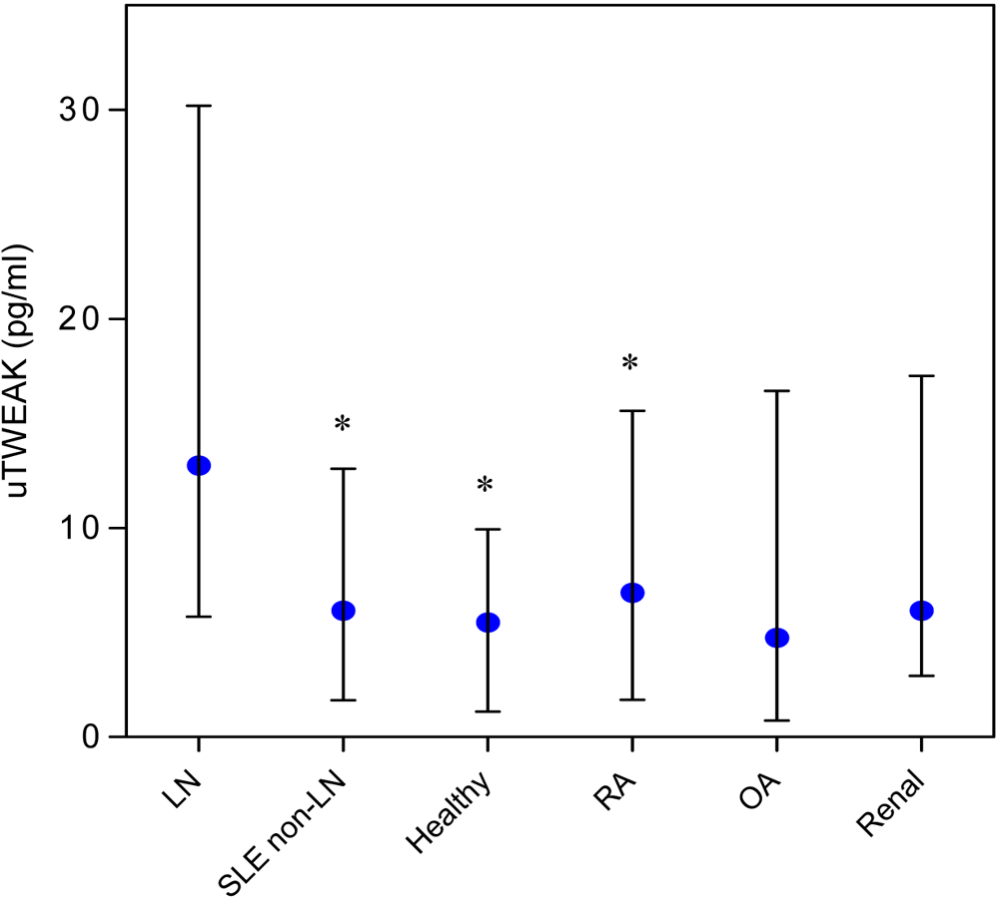
Systemic lupus erythematosus patients with lupus nephritis have high urinary TWEAK. A multigroup comparison between urinary TNF-like weak inducer of apoptosis (uTWEAK) levels of 30 patients with biopsy-proven lupus nephritis (LN), 49 systemic lupus erythematosus (SLE) patients without LN, 28 healthy individuals, 79 rheumatoid arthritis (RA) patients, 25 osteoarthritis (OA) patients and 31 renal controls (*P *= 0.039). *P < 0.05 in two-group post-hoc comparisons with LN. Graphs represent median levels with the interquartile range.

uTWEAK levels in each of the experimental groups were as follows: LN patients, 12.98 (5.76 to 30.19); SLE non-LN patients, 6.06 (1.77 to 12.83); normal controls, 5.48 (1.21 to 9.94); RA patients, 6.90 (1.79 to 15.61); OA patients, 4.75 (0.78 to 16.55); and renal patients, 6.04 (2.93 to 17.27). A multigroup comparison between uTWEAK levels of LN patients and the individual control groups yielded an overall significant difference (*P *= 0.039). Post-hoc testing revealed statistically significant lower uTWEAK levels in the SLE non-LN patient (*P *= 0.005), healthy control (*P *= 0.003), and RA patient (*P *= 0.013) groups, compared with the LN group (Figure 1).

Interestingly, renal disease *per se *did not independently raise uTWEAK levels, as there was no significant difference between uTWEAK levels of the renal controls compared with all other non-renal control groups (non-LN SLE patients, healthy controls, RA patients and OA patients) (6.04 (2.93 to 17.27) pg/ml vs. 5.85 (1.64 to 13.71) pg/ml, respectively; *P *= 0.313) - suggesting it is the combination of renal disease and SLE that leads to high uTWEAK levels. In addition, we found that high uTWEAK levels are a specific characteristic of LN and are not a general feature of SLE, as uTWEAK levels of non-LN SLE patients were not significantly different from those of the non-SLE controls (6.06 (1.77 to 12.83) pg/ml vs. 5.84 (1.84 to 14.26) pg/ml, respectively; *P *= 0.851).

### Urinary TWEAK differentiates between lupus nephritis and nonlupus nephritis systemic lupus erythematosus patients

**Figure 2:**
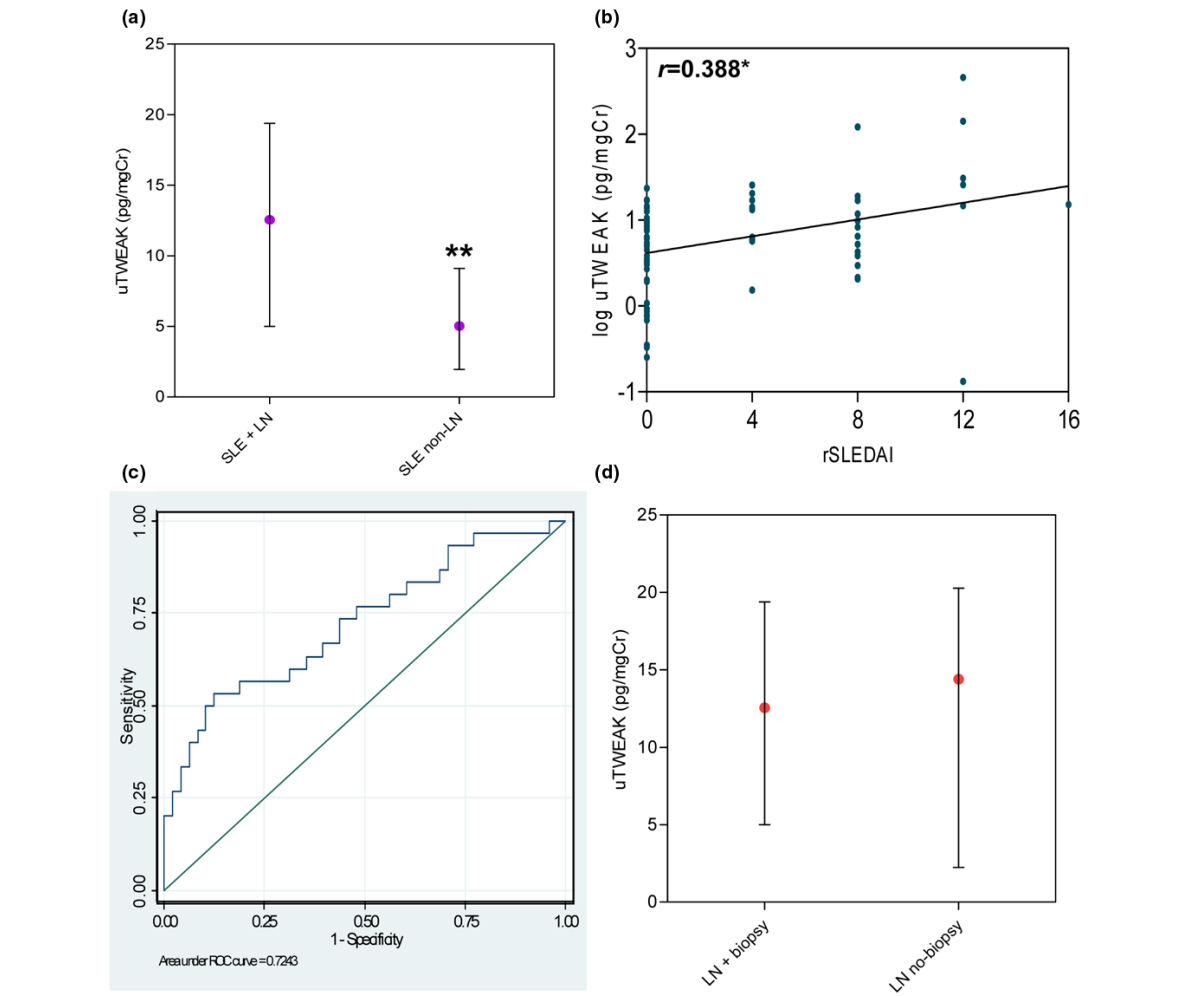
High urinary TWEAK is characteristic of lupus nephritis in systemic lupus erythematosus patients. **(a) **Thirty lupus nephritis (LN) patients have significantly higher urinary TNF-like weak inducer of apoptosis (uTWEAK) levels (corrected to creatinine) than 49 systemic lupus erythematosus (SLE) patients without LN (*P *< 0.001). **(b) **uTWEAK levels of 78 SLE patients (both LN patients and non-LN patients) correlate significantly with renal Systemic Lupus Erythematosus Disease Activity Index (rSLEDAI) scores (*r *= 0.388, *P *= 0.047). **(c) **Nonparametric receiver operating characteristic (ROC) curve for uTWEAK and the presence of LN in SLE patients; area under the curve = 0.724. **(d) **uTWEAK levels in 30 patients with biopsy-proven LN versus 11 SLE patients with a clinical diagnosis of LN not confirmed by renal biopsy (*P *= 0.941). Graphs represent median levels with the interquartile range. **P < 0.01.

We examined whether uTWEAK levels in LN patients remained significantly higher than that in non-LN SLE patients (using the same patients studied for Figure 1) even when uTWEAK is corrected to urinary creatinine. Indeed, when normalized to urinary creatinine, the median uTWEAK level of patients with active, biopsy-proven LN was 12.54 (5.00 to 19.38) pg/mgCr, while that of non-LN SLE patients was 5.02 (1.94 to 9.11) pg/mgCr (*P *< 0.001) (Figure 2a). As seen in Table 1, there is a significant difference in disease activity between the two SLE groups, since only patients with active renal disease (rSLEDAI ≥ 4) were included in the LN group. On the other hand, none of the non-LN patients, by definition, had any renal score in the SLEDAI, thus reducing the non-LN group's SLEDAI potential maximal score.

In an effort to isolate the effect of the nephritis on uTWEAK levels as opposed to general disease activity, we compared uTWEAK levels of LN patients and non-LN patients with an identical overall SLEDAI score of 4 (six LN patients and eight non-LN SLE patients). Under these circumstances, uTWEAK levels were also significantly higher in the LN group than in the non-LN group (13.60 (5.95 to 17.03) pg/mgCr vs. 2.77 (1.96 to 7.30) pg/mgCr, respectively; *P *= 0.008) - indicating that it is the nephritis and not systemic disease activity that raises uTWEAK levels. Furthermore, as shown in Figure 2b, and as similarly reported [16], it is the degree of activity of the renal disease that dictates uTWEAK levels, as a significant association was found between LN activity as measured by the rSLEDAI, and uTWEAK (*r *= 0.388, *P *= 0.047).

A nonparametric ROC curve, constructed to quantify how well uTWEAK distinguishes between SLE patients with or without nephritis, has an AUC of 0.724 (*P *< 0.001; Figure 2c). Interestingly, clinically used markers such as anti-dsDNA Ab, C3 and C4 within the same cohort of patients did not have the same discriminatory power (AUC of anti-dsDNA Abs = 0.599, *P *= 0.152; C3 = 0.577, *P *= 0.258; and C4 = 0.631, *P *= 0.053).

We dichotomized uTWEAK based on the above ROC curve, choosing a cutoff point of 13 pg/mgCr with a specificity of 90% and a sensitivity of 50%. We validated this cutoff point by testing its discriminating ability on a nonoverlapping cohort of patients described previously [16]. Indeed, using this cutoff point on the previous cohort we found a statistically significant association between patients being classified as having a high or low uTWEAK level and the presence of LN (*P *= 0.045). A logistic regression model adjusting for age, sex and ethnicity based on our current cohort showed an odds ratio of 7.36 (95% confidence interval = 2.25 to 24.07, *P *= 0.001) for high uTWEAK levels, correlating with the presence of LN.

To assess whether uTWEAK might be used to confirm a diagnosis of LN made based on clinical grounds alone, we compared uTWEAK levels of 30 patients with biopsy-proven LN and 11 SLE patients with a laboratory-based diagnosis of LN but with no confirming renal biopsy. In an attempt to ensure these latter patients indeed had LN and not just a concomitant renal abnormality, we only included patients with rSLEDAI scores ≥ 8 (requiring the presence of at least two out of the four abnormal renal disease parameters measured by the rSLEDAI). As predicted by our hypothesis, uTWEAK levels did not differ significantly between the two groups (*P *= 0.941; Figure 2d).

While high uTWEAK levels appear to be a feature of LN, no significant differences in uTWEAK were detected between different World Health Organization classes of glomerulonephritis (GN) found at biopsy (*P *= 0.412 in a comparison of classes III, IV and V).

### Urinary TWEAK is a marker of lupus nephritis activity

For the second part of the present study, we monitored and analyzed uTWEAK levels as a function of patient disease activity over time, including patients from both the AECOM and the OSS cohorts. As shown in Table 2, the analyzed patients from these two geographically distinct cohorts were relatively comparable, both in terms of their demographic parameters (age and sex) and of their disease activity (as measured by rSLEDAI scores, serum creatinine levels and World Health Organization class). The main difference between the groups was that the AECOM cohort also included patients without LN, and so the analysis included an appropriate adjustment for the presence of LN.

**Table 2:** Longitudinal study patient demographics

	AECOM cohort	OSS cohort	*P *value
Number of patients (% female)	31 (81)	13 (100)	0.091
Lupus nephritis (%)	58	100	0.005*
Number of visits per patient	3 (2 to 5)	3 (2 to 5)	0.508
Time between visits (months)	3 (2 to 6)	2 (2 to 6)	
Ethnicity^a^	18/12/0/1	5/0/8/0	
African American (%)	58	38	0.235
Age at first visit (years)	36 (31 to 44)	30 (28 to 36)	0.089
rSLEDAI^b^	4 (0 to 8)	4 (0 to 4)	
World Health Organization class^b^, *n *(%)			
III	6 (33)	1 (8)	0.092
IV	9 (50)	9 (69)	0.284
V	2 (11)	3 (23)	0.371
VI	1 (6)	0 (0)	0.388
Serum creatinine levels	0.8 (0.7 to 1.0)	1.0 (0.8 to 1.6)	
Only lupus nephritis patients	0.9 (0.7 to 1.1)		
At first visit^b^	0.8 (0.7 to 1.2)	1.03 (0.8 to 1.5)	0.160

Continuous variables presented as the median (interquartile range). AECOM, Albert Einstein College of Medicine; OSS, Ohio SLE Study; rSLEDAI, renal Systemic Lupus Erythematosus Disease Activity Index. ^a^African American/Hispanic/Caucasian/other.^b^Lupus nephritis patients only. *P < 0.05. Continuous variables were compared by the Mann- Whitney *U *test, while the two-sample proportion test was used for categorical variables. Statistical comparisons were not performed when values were not independent of each other.

**Figure 3:**
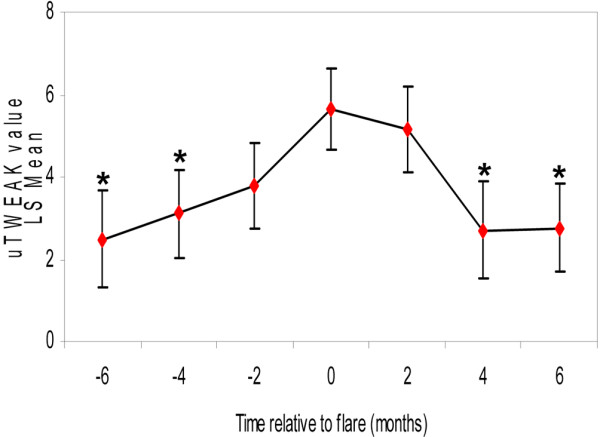
Urinary TWEAK levels at different timepoints relative to a lupus nephritis flare. Urinary TNF-like weak inducer of apoptosis (uTWEAK) levels of 13 lupus nephritis (LN) patients followed in the Ohio SLE Study cohort, taken 6, 4 and 2 months before and after a LN flare, including at the time of the flare itself (timepoint 0). Graph represents least-squares (LS) means with standard error. *P < 0.05 compared with uTWEAK level at flare.

The group of 13 patients from the OSS cohort was unique in that the patients had undergone a LN flare during their routine bi-monthly follow-up. Urine samples from before, during and after the flare were therefore prospectively collected. As shown in Figure 3, uTWEAK levels peaked during the flare, gradually increasing before and decreasing after the flare. Statistically significant differences were found between uTWEAK levels at flare as compared with 4 and 6 months before and after the flare timepoint (± 4 months, *P *= 0.035 and *P *= 0.025, respectively; ± 6 months, *P *= 0.017 for both timepoints). In addition, although the differences between uTWEAK levels at flare and at ± 2 months did not reach statistical significance, a clear trend in a similar direction is observed.

To analyze a larger sample of patients, we combined the longitudinal data of these 13 patients from the OSS cohort with data from a group of 31 unselected SLE patients from the AECOM cohort on whom serial measurements of uTWEAK levels had been obtained. (As most of these AECOM patients did not flare during the follow-up, these data were not included in the previous analysis.) A linear mixed-effects model using both the OSS and AECOM patient groups was constructed, with adjustments made for age, sex, ethnicity, and the presence of LN. This model showed a statistically significant association between uTWEAK levels of patients and their renal disease activity over time (β = 0.074, 95% confidence interval = 0.020 to 0.129, *P *= 0.008).

### Serum TWEAK levels and the urine/serum TWEAK ratio are not better markers than urinary TWEAK

A cross-sectional analysis of sTWEAK levels of SLE patients and controls was performed to test its biomarker function. To that end, available serum samples from 66 SLE patients (23 LN patients and 43 non-LN patients) and from 19 controls (healthy controls) from the AECOM cohort were analyzed for TWEAK levels. The sTWEAK data for 66 University of Southern California cohort SLE patients (35 LN patients and 31 non-LN patients) and for 20 healthy controls were also analyzed. Since the findings from both cohorts were similar, we present in detail only the data from the AECOM cohort (Table 3).

**Table 3:** Serum TWEAK results for the Albert Einstein College of Medicine cohort (pg/ml)

	Serum TWEAK	*P *value	AUC of ROC curve
SLE patients vs. healthy controls	15.87 (13.04 to 24.36), n = 66 vs. 23.56 (17.26 to 27.12), n = 19	0.034*	0.660
SLE + LN patients vs. SLE non-LN patients	16.42 (13.09 to 24.71), n = 23 vs. 15.72 (12.81 to 24.30), n = 43	0.747	-

Continuous variables presented as the median (interquartile range). LN, lupus nephritis; SLE, systemic lupus erythematosus The area under the curve (AUC) was calculated from nonparametric receiver operating characteristic (ROC) curves constructed to test the ability of serum TNF-like weak inducer of apoptosis (TWEAK) to distinguish between patients and healthy controls. *P < 0.05. The Mann- Whitney *U *test was used to compare between two groups.

The only significant finding in this cross-sectional analysis comparing sTWEAK levels of SLE patients and controls is that sTWEAK levels are significantly lower in SLE patients than in healthy individuals. Interestingly, similar findings that sTWEAK levels are lower in patients with renal disease than in healthy controls have been reported in the literature [22,23]. Nevertheless, this difference is not sufficient for sTWEAK to serve as a discriminating marker between healthy individuals and SLE patients, as the AUC of the constructed ROC curve was 0.660 and the sensitivity and specificity calculations were significantly inferior to those of anti-nuclear and anti-dsDNA Ab levels.

Since we found that uTWEAK is higher and sTWEAK is lower in SLE patients compared with healthy controls, we investigated whether the ratio of uTWEAK to sTWEAK may represent a better biomarker than uTWEAK alone. Nevertheless, while the uTWEAK/sTWEAK ratio performed similarly to uTWEAK alone in several analyses, it did not correlate significantly with cross-sectional disease activity as reflected by the rSLEDAI. Moreover, the AUC of the ROC curve testing the differentiating ability of the uTWEAK/sTWEAK ratio between LN patients and non-LN SLE patients was 0.673, worse than uTWEAK alone.

## Discussion

Recent findings have linked TWEAK to renal inflammation in an animal model of SLE [13,24], and on this premise we hypothesized that excreted uTWEAK may denote the presence and activity level of LN in SLE patients. Previously we determined that uTWEAK levels of LN patients are higher than those of non-LN SLE patients, and that uTWEAK levels correlate significantly with renal disease activity [16]. In our present study, high uTWEAK levels were found to reflect the presence of LN in SLE patients even better than clinical markers in widespread use, such as anti-dsDNA Ab and complement component levels. In fact, high uTWEAK levels in SLE patients correlate with sevenfold increased odds of LN. We observed that LN patients have higher uTWEAK levels than several other control groups analyzed, indicating that high uTWEAK levels are a relatively unique feature of LN and are neither due to the systemic inflammatory process (as occurs in non-LN SLE patients or in RA patients) nor the renal disease in isolation. Since the comparison of uTWEAK levels between the renal and nonrenal lupus groups in patients with identical SLEDAI scores did not include many patients in each group, however, it would be interesting to repeat this particular analysis in a larger study. Nevertheless, the correlation we found between uTWEAK levels and the severity of renal disease supports our conclusion that high uTWEAK levels in lupus patients primarily reflect renal activity. Furthermore, fluctuations of uTWEAK levels were found to reflect renal disease activity in LN patients over time, thus potentially serving as a helpful biomarker in the clinical follow-up.

It is interesting to consider whether any particular medication used in our patients may have contributed to variations in uTWEAK production. In previous work, however, we did not find any correlation between treatment with prednisone and other immunosuppressive drugs used to treat nephritis, and uTWEAK levels [16]. Nevertheless, this question is worthy of a more comprehensive analysis - in particular, focusing on whether effective therapy lowers uTWEAK levels over time. These studies are in progress. Finally, while in our study we did not find a difference in uTWEAK levels between the various World Health Organization stages of LN, particularly between the proliferative (class III, class IV) and membranous (class V) classes, this finding remains to be confirmed in larger numbers of patients displaying each of these histological subtypes.

The need for a reliable clinical biomarker for LN cannot be overstated. It is well established that long-term survival in SLE can be improved with early diagnosis and prompt treatment of the renal disease [25]. Nevertheless, the usually insidious onset and fluctuating nature of LN can make early identification and follow-up very difficult. While renal biopsy is the gold standard for the diagnosis and assessment of nephritis in lupus patients, it is an invasive procedure that is not generally performed serially for monitoring purposes. Anti-DNA Abs and complement levels are routinely followed in lupus patients but, while often correlating with the presence of active renal disease, these serologic parameters are not specific for this manifestation and their performance as nephritis biomarkers is not optimal [2,26-28]. In the absence of specific markers for LN, clinicians rely upon laboratory tests reflecting renal function, such as urinalysis, urinary protein measurements, blood urea nitrogen and serum creatinine. These measurements are useful in monitoring chronic renal processes, but abnormal levels may occur relatively late in the inflammatory course.

A number of potential biomarkers have been described in the past few years, although none has yet been validated [26]. Among the more promising suggested biomarkers are the chemokines inducible protein 10 (IP-10) and monocyte chemoattractant protein 1 (MCP-1). Active SLE patients were shown to have increased levels of IP-10, as opposed to nonactive SLE patients, RA patients and healthy controls [29,30]. Moreover, it has been reported that IP-10 mRNA levels isolated from urine cells can distinguish diffuse proliferative GN from other classes of LN [31]. MCP-1 has also been implicated in the pathogenesis of LN and suggested as a potential biomarker by Rovin and colleagues, who demonstrated that urinary levels of MCP-1 may predict impending flare, flare severity and response to treatment. Similar to our findings regarding uTWEAK, however, MCP-1 was not reported to predict renal histopathology [17]. Of note, both IP-10 and MCP-1 are among the proinflammatory chemokines induced by TWEAK in mesangial [13,32] and tubular [33] renal cells.

In our longitudinal study, although uTWEAK levels did increase as the flare approached, the peak was at the time of the flare rather than in advance of it. As the measurements were based on a relatively small group of 13 patients, however, it is possible that there was simply not enough power to be conclusive on this point. While under the limitations of our study TWEAK was not found to be predictive of the flare, an increase in uTWEAK levels at the time of the flare may therefore be able to provide supporting evidence if the diagnosis of a flare is in doubt.

uTWEAK levels of biopsy-proven LN patients were not different from those of patients diagnosed clinically with LN yet were significantly higher than those of non-LN patients, implying that high uTWEAK levels support the diagnosis of LN - perhaps circumventing the need in some patients for a diagnostic, yet invasive, biopsy. Nevertheless, uTWEAK levels do not distinguish between different World Health Organization GN classes among LN patients, and therefore cannot entirely replace kidney biopsy in the diagnostic process.

Certain limitations encountered in our study should be addressed in the future, foremost of which is our inability to compare the performance of uTWEAK with proteinuria, the traditional measure that is used. As our definition of LN was based on proteinuria, we could not examine proteinuria as an independent marker in comparison with uTWEAK. Future studies including an additional disease activity index - such as the British Isles Lupus Assessment Group (BILAG) [34], for example- would be a means of further validating the presented results before clinical application.

A potential role for TWEAK in LN has been explored experimentally *in vitro *and *in vivo*. As mentioned, TWEAK has been found to induce secretion of known chemokine mediators of SLE, such as MCP-1, IP-10 and RANTES [13,33]. In addition, TWEAK/Fn14 interaction induces prolonged NF-κB activation in human renal mesangial cells [32], and it is now thought that prolonged activation of this route underlies chronic inflammatory diseases [15]. Specific to LN, Zhao and colleagues demonstrated that TWEAK plays an important role in the murine chronic graft versus host model of LN, such that Fn14-knockout mice, or mice treated with anti-TWEAK monoclonal antibodies, exhibited reduced inflammation and less severe nephritis [24].

Since uTWEAK but not other urinary proteins correlate with renal disease [16], and since urine but not serum levels are elevated, high uTWEAK levels seem to reflect high local renal production rather than increased total urine protein. Moreover, in our study uTWEAK levels were clearly not a reflection of renal function, since uTWEAK levels in patients with LN were higher than in non-LN patients with both significantly higher (renal disease group) and lower (non-LN SLE group) serum creatinine levels (Figure 1). Accordingly, the increased levels of uTWEAK and their correlation with renal disease activity over time represent increased renal expression of TWEAK at the time of the nephritis process, providing additional evidence for the role of TWEAK in the pathogenesis of LN.

## Conclusions

The relapsing- remitting course of LN, among the most serious complications of SLE, requires close monitoring and often frequent treatment adjustments throughout patients' lives. Kidney biopsy, however, is impractical as a clinical surveillance tool. A dependable biomarker that can reflect the patient's renal disease activity is therefore highly desirable. We show here that uTWEAK can serve as a biomarker of LN, both as a one-time measurement and as a means of monitoring individual patients over time. Moreover, uTWEAK performed significantly better than traditional biomarkers in widespread use (anti-dsDNA Abs, C3 and C4) in distinguishing between lupus patients with and without nephritis. Analysis of uTWEAK levels in an expanded panel of patients followed longitudinally with particular focus on measurements prior to clinical evidence of disease activity as well as correlations with patients' response to a given therapy, together with measurements of other emerging biomarkers, will help define a role for the serial measurement of uTWEAK levels in the clinical management of lupus patients with suspected or existing renal involvement. Finally, uTWEAK levels may also be useful in the future to identify potential candidates for therapies intended to block the TWEAK signaling pathways [8,24].

## Abbreviations

AECOM: Albert Einstein College of Medicine; anti-dsDNA Ab: anti-double stranded DNA antibody; AUC: area under the curve; BSA: bovine serum albumin; ELISA: enzyme-linked immunosorbent assay; GN: Glomerulonephritis; IP-10: inducible protein 10; LN: lupus nephritis; MCP-1: monocyte chemoattractant protein 1; NF: nuclear factor; OA: osteoarthritis; OSS: Ohio SLE Study; PBS: phosphate-buffered saline; *r*: Spearman rank correlation coefficient; RA: rheumatoid arthritis; ROC: receiver operating characteristic; rSLEDAI: renal Systemic Lupus Erythematosus Disease Activity Index; SLE: systemic lupus erythematosus; SLEDAI: Systemic Lupus Erythematosus Disease Activity Index; sTWEAK: serum TNF-like weak inducer of apoptosis; TNF: tumor necrosis factor; TWEAK: TNF-like weak inducer of apoptosis; uTWEAK: urinary TNF-like weak inducer of apoptosis.

## Competing interests

LCB, LS, and JSM are full-time employees and have stock ownership or options in Biogen Idec. CP received grant support from Biogen Idec. LCB, JSM and CP have a patent application under review describing the use of TWEAK measurements. The remaining authors declare that they have no competing interests.

## Authors' contributions

NS, TR, LCB, CEC, IB, WS, BHR, JSM, and CP contributed to the study design, and to interpretation and analysis of the data. NS, LCB, JSM, and CP prepared the manuscript. LS performed the ELISA assays. NS, TR, CEC, IB, BH, MM, and CA contributed to the sample collection and data acquisition.
